# CA-CAS-01-A: A Permissive Cell Line for Isolation and Live Attenuated Vaccine Development Against African Swine Fever Virus

**DOI:** 10.1007/s12275-024-00116-1

**Published:** 2024-03-13

**Authors:** Seung-Chul Lee, Yongkwan Kim, Ji-Won Cha, Kiramage Chathuranga, Niranjan Dodantenna, Hyeok-Il Kwon, Min Ho Kim, Weonhwa Jheong, In-Joong Yoon, Joo Young Lee, Sung-Sik Yoo, Jong-Soo Lee

**Affiliations:** 1 Choong Ang Vaccine Laboratories, Daejeon, 34055 Republic of Korea; 2 Wildlife Disease Response Team, National Institute of Wildlife Disease Control and Prevention, Gwangju, 62407 Republic of Korea; 3 College of Veterinary Medicine, Chungnam National University, Daejeon, 34134 Republic of Korea

**Keywords:** CA-CAS-01-A (CAS-01), African swine fever virus (ASFV), Virus adaptation

## Abstract

African swine fever virus (ASFV) is the causative agent of the highly lethal African swine fever disease that affects domestic pigs and wild boars. In spite of the rapid spread of the virus worldwide, there is no licensed vaccine available. The lack of a suitable cell line for ASFV propagation hinders the development of a safe and effective vaccine. For ASFV propagation, primary swine macrophages and monocytes have been widely studied. However, obtaining these cells can be time-consuming and expensive, making them unsuitable for mass vaccine production. The goal of this study was to validate the suitability of novel CA-CAS-01-A (CAS-01) cells, which was identified as a highly permissive cell clone for ASFV replication in the MA-104 parental cell line for live attenuated vaccine development. Through a screening experiment, maximum ASFV replication was observed in the CAS-01 cell compared to other sub-clones of MA-104 with 14.89 and log_10_ 7.5 ± 0.15 Ct value and TCID_50_/ml value respectively. When CAS-01 cells are inoculated with ASFV, replication of ASFV was confirmed by Ct value for ASFV DNA, HAD_50_/ml assay, TCID_50_/ml assay, and cytopathic effects and hemadsoption were observed similar to those in primary porcine alveolar macrophages after 5th passage. Additionally, we demonstrated stable replication and adaptation of ASFV over the serial passage. These results suggest that CAS-01 cells will be a valuable and promising cell line for ASFV isolation, replication, and development of live attenuated vaccines.

## Introduction

African swine fever virus (ASFV) causes the highly contagious disease, African swine fever (ASF), which results in high mortality rates in pigs. Early reports of ASF in Kenya in 1921 were associated with an ancient sylvatic cycle, which caused nearly 100% mortality in domestic pigs infected with acute hemorrhagic fever (Monteagudo et al., 2017). Following its introduction to Portugal from Angola in 1957, it spread across Europe and caused significant losses to the pig industry. However, European countries, except Sardinia, succeeded in eradicating the disease in 1995 through stringent disease control measures (Chathuranga & Lee, 2023; Martins et al., 2021; Turlewicz-Podbielska et al., 2021). A number of transcontinental occurrences have been reported since 2007, with the most significant occurrence in Georgia in 2007. Since then, other transcontinental occurrences have been reported in Africa, Europe, Asia, and Oceania and were most recently introduced in North America (Sun et al., 2021). The World Organization for Animal Health documented a span from January 2020 to January 2022 during which ASF outbreaks were observed across 35 different countries or regions on a global scale. These outbreaks resulted in the infection of 4767 domestic pigs, leading to the unfortunate loss of 1,043,334 animals, as well as 18,262 cases in wild boars, causing the loss of 29,970 animals (Wang et al., 2022). The causative pathogen responsible for ASF is a sizeable double-stranded DNA virus classified within the Asfaviridae family (Cackett et al., 2020). Robust biosecurity measures and established sanitary practices implemented both at the farm and national scales have been employed in the ongoing effort to combat the dissemination of this virus. Nonetheless, the effectiveness of these approaches remains limited, particularly in certain regions where resource constraints pose challenges to their full implementation. Therefore, there is an urgent need to develop an effective ASFV vaccine. Over the past few decades, numerous vaccination tactics have been explored, encompassing approaches such as inactivated vaccines, DNA-based vaccines, subunit vaccines, and viral-vector-based vaccines. However, most of these vaccines failed to induce effective protective immunity against ASFV (Chathuranga & Lee, 2023). Through the culmination of prior investigations, the most encouraging vaccine candidates to date consist of live attenuated African Swine Fever viruses (LA-ASFV), demonstrating remarkable efficacy, offering complete protection of up to 100% against homologous pathogenic ASFV challenges (Pérez-Núñez et al., 2022; Tran et al., 2022). Live attenuated viruses have reduced virulence as a result of deletion of virulence-associated genes, either naturally (King et al., 2011) or through passage into tissue culture (Krug et al., 2015) or through genetic modification methods (Abkallo et al., 2021; Borca et al., 2018). However, for the development of an effective ASFV vaccine using LA-ASFVs, specific cells capable of producing stable ASFV vaccine strains are essential.

Pig-derived ASFV isolates have restricted cell tropism and normally replicate only in primary porcine cells such as blood-derived macrophages, monocytes, and pulmonary alveolar macrophages. Hence, primary monocytes or alveolar macrophages have been employed to investigate the isolation and amplification of ASFV, explore virus–host interactions, and replicate ASFV infections in in vivo models (Franzoni et al., 2017). However, primary cells have disadvantages including low reproducibility, high batch-to-batch variation, time-intensive procedures, expensive cell extraction, and animal welfare considerations (Meloni et al., 2022). Hence, it is imperative to discern passaged cell lines that facilitate robust ASFV replication, facilitating its isolation, virus purification, aiding biological investigations, and enabling the development of live attenuated vaccines. To date, limited ASFVs were propagated, titrated, and passaged using several established cell lines such as IPAM, COS-1, WSL, Vero, and PIPEC (Carrascosa et al., 2011; Meloni et al., 2022). However, there are no commercially available cell lines that have been shown to be suitable for passaging from field samples to produce LAVs. In this study, we derived and validated highly ASFV permissive homogenous cell clone from MA-104 parental cell line, denoted as CA-CAS-01-A (CAS-01) and validated for ASFV replication and stable virus passage.

## Materials and Methods

### African Swine Fever Virus Isolation

**Fig. 1:**
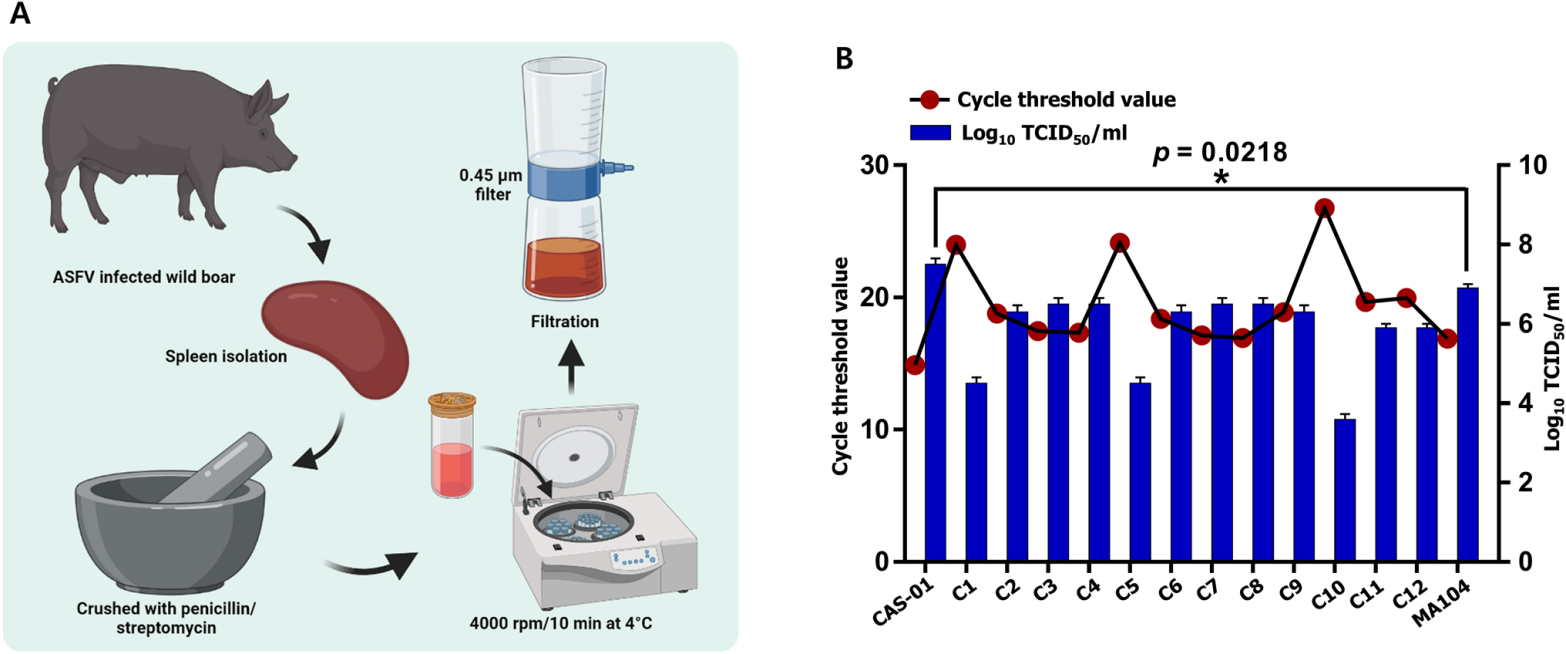
Isolation of African swine fever virus and screening of MA-104 cell subpopulations against ASFV infection. **A** ASFV-positive wild boar spleen samples, delivered from the National Institute of Wildlife Disease Control and Prevention (NIWDC) were crushed in PBS with 1% penicillin/streptomycin (P/S), and the crushed tissues were centrifuged at 4 °C and 4000 rpm for 10 min. Then, the supernatant was filtered through a 0.45 μm filter. The obtained virus was stored at − 80 °C for further experiments. **B** ASFV replication in different cell clones. Virus replication in different MA-104 cell clones and parental cell lines was evaluated by real-time PCR and TCID_50_ assay at 6 dpi at the 7th passage in each clone. TCID_50_ results expressed as mean ± SD and *p < 0.05 regarded as a significant difference

African swine fever virus (ASFV) positive spleen samples from wild boar were provided by the National Institute of Wildlife Disease Control and Prevention (NIWDC) in Republic of Korea. Next, the spleen sample was immersed in PBS supplemented with 1% penicillin/streptomycin (P/S), and minced. The mashed tissue was centrifuged at 4 °C and 4000 rpm for 10 min, and the supernatant was separated. Then, the separated supernatant was filtered through a 0.45 µm filter. Afterward, viral DNA extraction was performed from the filtered suspension, and virus positivity was reconfirmed through real time polymerase chain reaction (RT-PCR), and used immediately in the experiment or stored at − 80 °C (Fig. 1A). The two ASFV field strains used in this study was ASFV/INJE/11893 and ASFV/INJE/13167/2021. All experiments dealing with ASFV were conducted in accordance with the Standard Operating Procedure (SOP) in the biosafety level 3 (BSL-3) laboratory of the NIWDC in Korea.

### Cell Lines and CAS-01 Cell Cloning

Primary pulmonary alveolar macrophage (PAM) (Optipharm Inc.) was cultured in 10% Fetal bovine serum (FBS) (Gibco™) and 1% Penicillin–Streptomycin (Gibco™) added RPMI-1640 medium (Hyclone™) in an incubator at 37 °C and 5% CO_2_ atmosphere. African green monkey kidney epithelial cell line (MA-104, CRL-2378.1TM) was cultured in Alpha Modification of Eagle's Minimum Essential Media (Gibco™) supplemented with 10% FBS and 1% Penicillin–Streptomycin at 37 °C with 5% CO_2_ environment. Cloning of the MA-104 cell subpopulations was performed by the limiting-dilution method. Suspended MA-104 cells were diluted at a mean concentration of 1 cell/well in 10% FBS containing MEM media and dispensed in to 96-well cell culture plates. Cells were incubated at 37 °C in an atmosphere of 5% CO_2_. The initial monolayer of cell cloning was subjected to subsequent sub-cloning for cell amplification. In order to select high-permissive cell clones for ASFV, cell clones were infected with ASFV in to each monolayer for seven passages. Virus replication was determined at the 7th passaged virus by the tissue culture infectious dose (TCID_50_) and RT-PCR assays. MA-104 cell line was used as the reference, and the cell clone with higher ASFV replication was further amplified, stocked and denoted as CA-CAS-01-A (CAS-01). CAS-01 cells (Choong Ang Vaccine Laboratories Co., Accession No. KCTC 14568BP) were cultured in Alpha Modification of Eagle's Minimum Essential Media (Gibco™) supplemented with 10% FBS and 1% Penicillin–Streptomycin at 37 °C with 5% CO_2_ atmosphere.

### Virus Infection and ASFV Passage in CAS-01

CAS-01 cells were cultured at 5 × 10^5^ cells/flask in a T25 flask and incubated in 2% FBS and 1% Penicillin–Streptomycin. Next, isolated ASFV/INJE/11893 and ASFV/INJE/13167/2021 was infected into cells separately, and culture media was replaced with virus production-serum free medium (VP-SFM) (Gibco™). Twenty-four hours later, again media were changed with VP-SFM added 2% FBS and incubated for 6 days in a 5% CO_2_ atmosphere at 37 °C. The collected cell pellet was subjected to two-time freezing and thawing (F/T), and it was re-suspended with the supernatant. Then, 2 ml of the suspension was continuously passaged in a similar manner to the above. Cycle threshold value (Ct value) was measured on the 7 days of post-infection (dpi) for each passage, and viral infectivity was confirmed by staining through Immunocytochemistry (ICC) using immunoperoxidase assay.

### Extraction of Viral DNA

Three hundred microliter of isolated ASFV virus or ASFV-infected cell lysate was mixed with 200 μl of cell lysis buffer and 20 μl of Proteinase K (Promega™, MC5005). Next, the mixture was heated at 56 °C for 30 min. Viral DNA was extracted in 50 μl increments using the Maxwell^®^ RSC 48 Instrument in the Maxwell RSC Whole Blood DNA Kit (Promega, AS1520) according to the manufacturer's instructions.

### Real-Time Polymerase Chain Reaction (RT-PCR)

The Ct value for the ASFV DNA template was confirmed using the commercially available VetMAX™ African Swine Fever Virus Detection Kit (ThermoFisher™, A28809) targeting the ASFV P72 gene (B646L). RT-PCR was performed in the QuantStudio™ 6 Flex Real-Time PCR (Applied Biosystems™, 4485691) machine using the Quant Studio Real-Time PCR Software according to the manufacturer's instructions for a total of 40 cycles.

### Immunocytochemistry (ICC) Using Immunoperoxidase Assay

ASFV-infected CAS-01 cells were subjected to ICC assay. In order to fix the cells, 80% Acetone was added on the cells after removing the media from the plate. The cells were allowed to dehydrate and fix for 10 min after adding the acetone. Afterward, acetone was removed and 10 min of drying were undertaken at room temperature. Next, cells were washed once with PBS, and rabbit Anti-ASFV p30 polyclonal antibody (Creative-diagnostics™, CABT-RM033) was added as the primary antibody at a 1:500 dilutions and incubated at 37 °C for 1 h. Following 4 times washing with PBS, polyclonal goat anti-rabbit immunoglobulin (Enzo™, ADI-SAB-301-J) was used as a secondary antibody at a 1:1000 dilution and incubated at 37 °C for 1 h. Finally, cells were washed 4 times with PBS and detected using alkaline phosphatase substrate (ImmPACT^®^ Vector^®^ Red Substrate, Alkaline Phosphatase, Vector, SK-5100). The substrate was washed aware with distilled water to avoid over-staining.

### Determination of Viral Titers by Hemadsorption Doses (HAD_50_)

Primary PAM cells were seeded at 5 × 10^5^ cells/ml respectively and incubated under 37 °C temperature and 5% CO_2_ atmosphere. Serial dilution of the ASFV-infected sample was prepared in 96-well U bottom plate. Twelve hours later, Primary PAM cells were infected with tenfold diluted ASFV-containing samples. At 2-h post-infection (2 hpi), 0.4% red blood cell (RBC) was added to each well and incubated for 7 days. The wells showing hemadsorption were marked every day for 7 days. Finally, using the reed-Muench method, wells showing hemagglutination were selected and counted.

### Determination of Viral Titers by Tissue Culture Infectious Dose (TCID_50_)

CAS-01 was incubated at 1 × 10^5^ cells/ml in a 96-well plate at 37 °C and 5% CO_2_ atmosphere. After 12 h of cell incubation, ASFV was serially diluted in a 96-well U bot-tom plate to infect the cells and cultured in 96 well-flat bottom plates for 7 days. The wells showing virus-induced CPE were marked every day for 7 days. Then, using the Reed and Muench method, wells showing CPE were selected and calculated. An immunoperoxidase assay was performed on the plate on which CPE was confirmed. Tissue Culture Infectious Dose was calculated using the Reed and Muench method by counting wells in which more than 50% of the cells inside the wells were stained.

### Statistical Analysis

The statistical analysis was performed using GraphPad Prism software version 6 for Windows (GraphPad Software). Comparison between CAS-01 and MA-104 cell TCID_50_ results were analyzed by the unpaired t-test. *p < 0.05 was regarded as statistically significant.

## Results

### Identification of ASFV Permissive Homogeneous Cell Subpopulation from MA-104 Parental Cell Line

Recently, two research groups identified MA-104 cells as an ASFV susceptible cell line (Kwon et al., 2022; Rai et al., 2020). However, isolated focal cytopathic effects were also observed in the ASFV-infected MA-104 monolayers, emphasizing the presence of highly ASFV-permissive MA-104 cell subpopulations in the heterogeneous parental MA-104 cells. In a previous study, a homogenous porcine reproductive and respiratory syndrome (PRRS)-susceptible cell population was identified and isolated from MA-104 cells and named MARC-145 (Kim et al., 1993). In this study, isolated MA-104 subpopulations were infected with ASFV for seven passages, and ASFV replication at 7th passage was evaluated by real-time PCR (RT-PCR) for ASFV DNA and TCID_50_ assays. As shown in Fig. 1B, we found that susceptibility to ASFV infection was different in each cell clone compared to the MA-104 parental cell line. Finally, we identified a clone with higher viral replication than that of the parental cell line, denoted as CA-CAS-01-A (CAS-01) cell. CAS-01 cells were deposited in the Korean Collection for Type Cultures (KCTC) under accession no. KCTC 14568BP.

### Isolation and Adaptation of ASFV in CAS-01 Cells

**Fig. 2:**
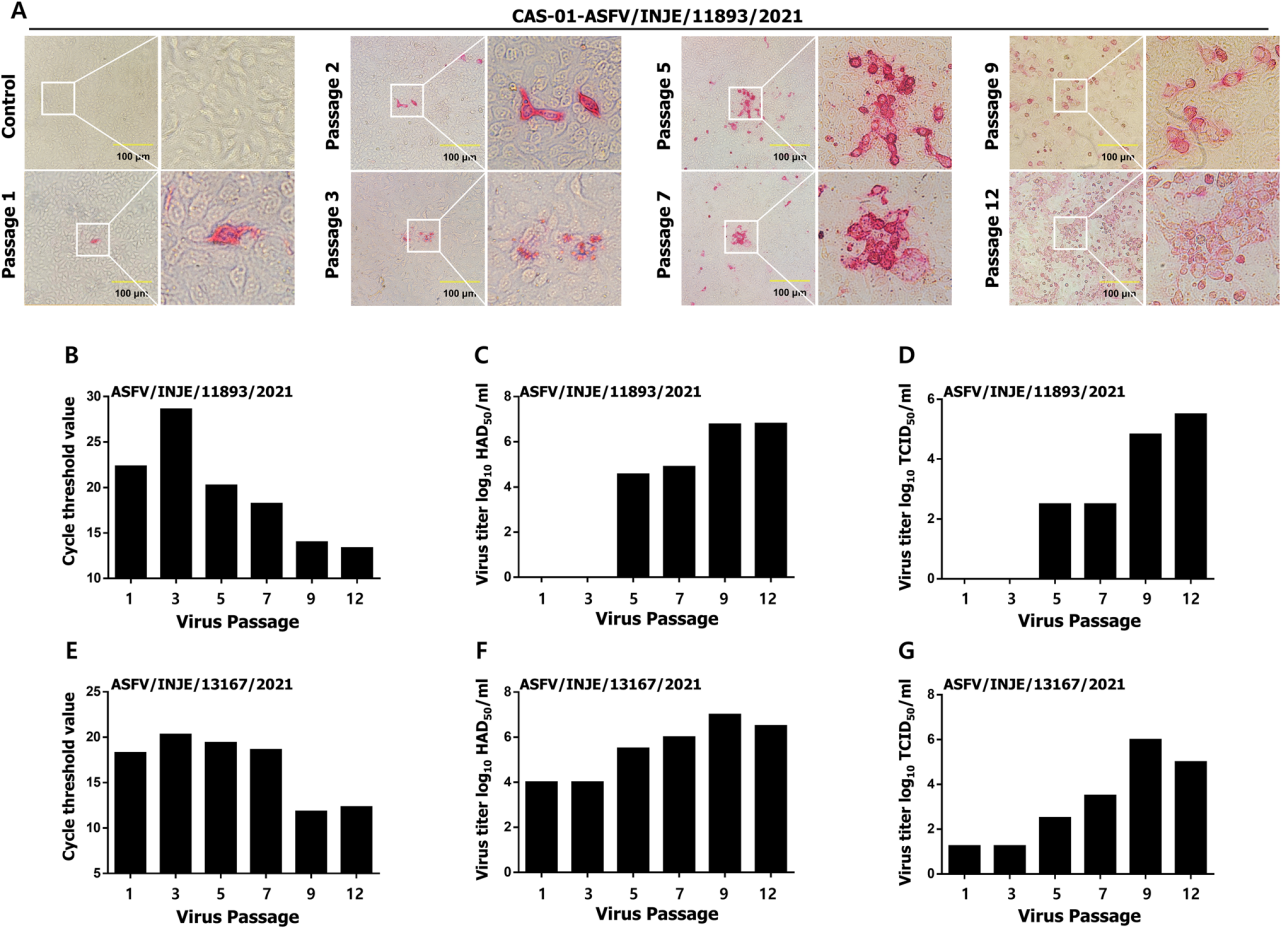
Adaptation of ASFV in CAS-01 cells. The ASFV/INJE/11893/2021 or ASFV/INJE/13167/2021 virus was infected with CAS-01 cells at 1 × 10^5^ cells/flask in a T25 flask and incubated in 2% FBS and 1% P/S added VP-SFM. After 24 h, media was changed by VP-SFM added 2% FBS, and incubated 6 days in a 5% CO_2_ atmosphere at 37 °C. **A**–**D** ASFV/INJE/11893/2021, **E**–**G** ASFV/INJE/13167/2021 infection results are shown. **A** Immunocytochemistry (ICC) analysis using immunoperoxidase assay with ASFV p30 antibody for indicated ASFV passages to detect the ASFV infectivity. **B**, **E** Ct value was measured on the 7 dpi for each passage. The replicated viruses were titrated using **C**, **F** HAD_50_ and **D**, **G** TCID_50_ in the indicated ASFV passages

To determine the CAS-01 cell susceptibility to ASFV infection, and also to observe the susceptibility of CAS-01 cells with ASFV passaging, field isolated two strains, ASFV/INJE/11893 and ASFV/INJE/13167/2021 was passaged for 12 consecutive passages in CAS-01 cells. In immunocytochemistry assays, enhanced p30 alkaline phosphatase staining was observed with passaging, but not in control CAS-01 cells at 24 hpi (Fig. 2A). Moreover, we observed enhanced grape-like clusters of CAS-01 cells, with a round morphology, and cytopathic effects of the cells with passaging of ASFV, which positively correlated with higher viral propagation (Fig. 2A). (Given data was obtained from ASFV/INJE/11893 strain infection). However, as shown in the ICC assay results, the level of detectable p30 protein was low in early passages, but it was robustly enhanced in late passages. To validate the results observed during the ICC assay, an RT-PCR assay to detect ASFV DNA, as well as HAD_50_ and TCID_50_ assays were performed for the cells or viruses collected during each passage. During the serial passaging of ASFV/INJE/11893/2021, the Ct value of ASFV DNA gradually increased from passage 1 to the 3rd passage (21.703, and 28.608 at passages 1, and 3, respectively). However, we observed a rapid de-crease in the Ct value from the 5th passage to the 12th passage, exhibiting rapid viral propagation (20.653, 18.236, 13.988, and 13.348 at passages 5, 7, 9, and 12, respectively) (Fig. 2B). Furthermore, during serial passaging of ASFV, the viral titers in CAS-01 cells increased and stably maintained the virus replication with increasing passages as of the 5th passage, reaching a peak at the 12th passage, with log_10_ 6.8 HAD_50_/ml and log_10_ 5.5 TCID_50_/mL (Fig. 2C, D). As shown in Fig. 2A, the infectivity of isolated ASFV was verified in the initial passage through the ICC staining assay, and hemadsorption was distinctly confirmed in early passages, however, it remains uncertain whether it surpasses 50% of the total wells. Thus, we were unable to obtain the TCID_50_ and HAD_50_ results from passages 1 and 3. Interestingly, ASFV/INJE/13167/2021 infection results Ct value of ASFV DNA 18.300 in 1st passage while virus replication was detected as 4.0 HAD_50_/ml and log_10_ 1 TCID_50_/ml. Similar to the ASFV/INJE/11893/2021 strain infection ASFV/INJE/13167/2021 serial passage exhibited a rapid increase of virus replication (Fig. 2E–G). Together, our data demonstrate that ASFV infection could detected from the first passage via ICC, virus titer and hemadsorption was distinctly confirmed in CAS-01 cells, while significantly higher virus replication was detected via TCID_50_ and HAD_50_ assays from passaged virus, indicating the potential of employing this novel cell line for ASFV isolation, replication, and adaptation.

### Replication and Characteristics of ASFV in CAS-01 Cells

**Fig. 3:**
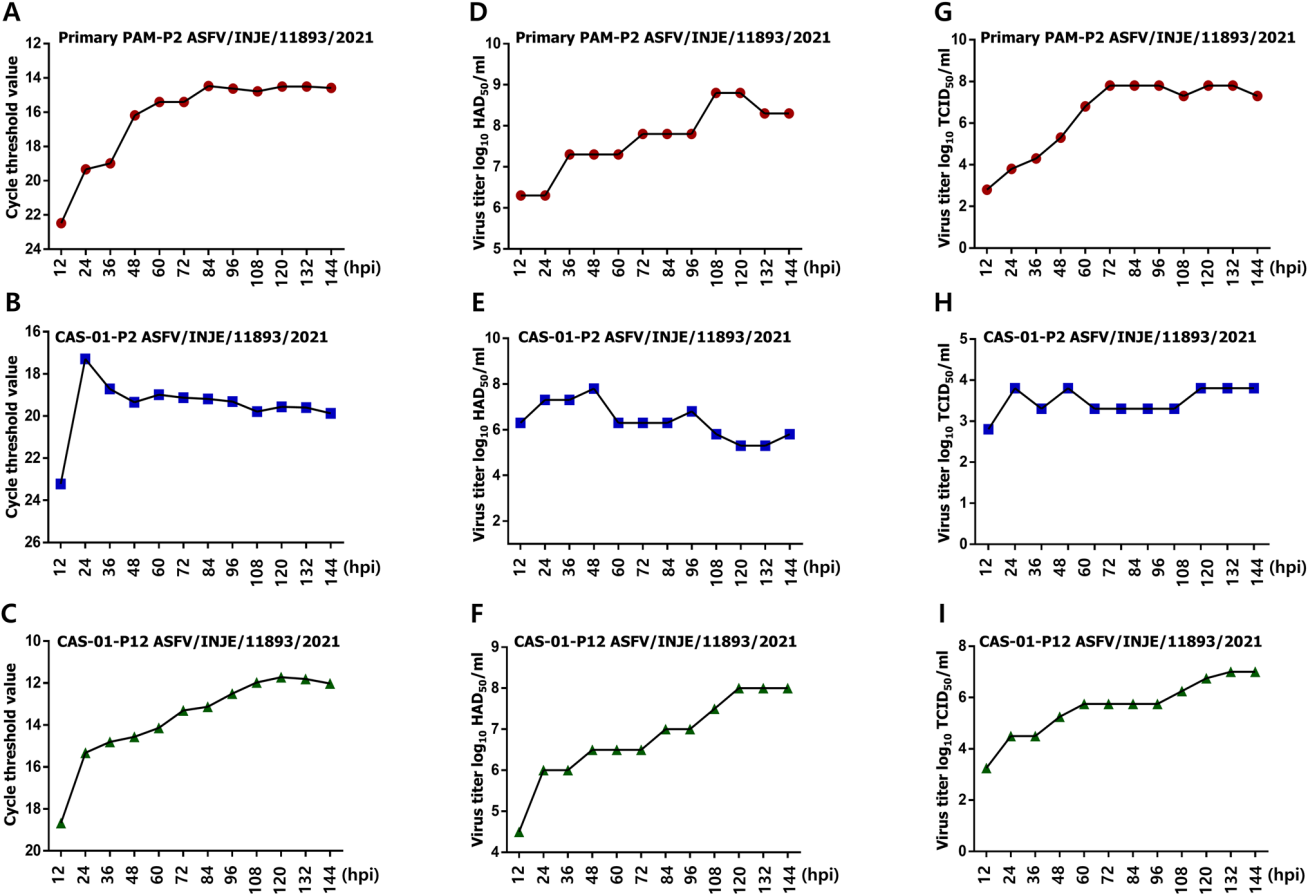
Growth kinetics of ASFV in Primary PAM and CAS-01 cell. ASFV/INJE/11893/2021 was serially passaged twelve times in CAS-01 cells. Primary PAM (**A**, **D**, **G**) and CAS-01 cells (**B**, **C**, **E**, **F**, **H**, **I**) were incubated at 5 × 10^5^ cells/well or 1 × 10^5^ cells/well, respectively in a 6 well plate at 37 °C and 5% CO_2_ atmosphere. After 12 h of cell incubation, cell passaged P-2 or P-12 ASFV was infected using VP-SFM with 1% FBS and 1% S/P. Cells were harvested at 12-h intervals up to 144 h after virus infection. After freezing and thawing (F/T) twice, Ct values **A**–**C** were obtained using RT-PCR. Viruses were titrated using **D**–**F** 50% hemadsorption doses (HAD_50_) and **G**–**I** 50% tissue culture infectious dose (TCID_50_)

Viral growth kinetics were determined in both primary PAM and CAS-01 cells using RT-PCR, HAD_50_, and TCID_50_ assays to assess the efficiency of ASFV infection over time. Both cell lines were infected with cell passaged P-2 or P-12 ASFV for 2 h, and virus replication was evaluated at the indicated time points using different methods. The cycle threshold (Ct) value results of P-2 ASFV DNA in primary PAM cells were 22.474, 19.331, 18.983, 16.18, 15.405, 14.471, 14.622, 14.79, 14.508, 14.508, 14.581 (Fig. 3A), and the Ct value results of P-2 ASFV DNA in CAS-01 cells were 23.221, 17.288, 18.716, 19.346, 18.988, 19.129, 19.185, 19.315, 19.786, 19.56, 19.593 and 19.952 at 12, 24, 36, 48, 60, 72, 84, 96, 108, 120, 132, and 144 hpi respectively (Fig. 3B). Interestingly Ct value results of P-12 ASFV DNA was 18.667, 15.325, 14.807, 14.561, 14.138, 13.308, 13.128, 12.507, 11.971, 11.722, 11.805, and 12.023 at 12, 24, 36, 48, 60, 72, 84, 96, 108, 120, 132, and 144 hpi respectively (Fig. 3C). As indicated by the Ct values, P-2 ASFV replication peaked in CAS-01 cells at 24 hpi. The viral titer remained stable until 144 hpi but indicated lower viral replication than that of primary PAM cells. However, P12-ASFV infection in CAS-01 cells resulted in significantly lower Ct values than P-2 ASFV in CAS-01 cells, reflecting the higher virus replication of the P12 virus in CAS-01 cells. To further evaluate the potential of CAS-01 cells to support ASFV replication, we compared the viral titers by HAD_50_ and TCID_50_ in primary PAM and CAS-01 cells. As shown in Fig. 3D, P-2 ASFV-infected primary PAM cells exhibited a log_10_ 6.3010 HAD_50_ value at 12 hpi and viral propagation continued until 144 hpi. In contrast, at 12 hpi, CAS-01 cells had a log_10_ of 6.3011 HAD_50_/ml value and reached a maximum at 48 hpi, with a log_10_ of 7.800 HAD_50_. However, the HAD_50_ value fluctuated from 60 to 144 hpi. Thereafter, the viral production started to decrease slightly at 144 hpi (Fig. 3E). Interestingly, P12-ASFV infection in CAS-01 cells exhibited a log_10_ 4.499 HAD_50_ value at 12 hpi, and viral propagation continued until 144 hpi (log_10_ 8.000 HAD_50_) (Fig. 3F).

Next, we assessed and compared the propagation of ASFV in primary PAM and CAS-01 cells by TCID_50_ assay. The kinetics of P-2 ASFV replication in primary PAM cells showed that virus production increased from 12 hpi, reaching a maximum titer of log_10_ 7.3010 TCID_50_/ml at 144 hpi (Fig. 3G). On the other hand, the kinetics of P-2 ASFV replication in CAS-01 cells showed that virus propagation increased from 12 hpi, reaching a maximum titer of log_10_ 3.800 TCID_50_/ml at 48 hpi, after which viral production started to decrease slightly and fluctuate until 144 hpi (Fig. 3H). Because the early-passaged ASFV had low virus concentration, the virus titer was highest at 48 hpi and comparatively lower than that of primary PAM cells at the tested time points. However, as shown in Fig. 3I, kinetics of P-12-ASFV replication in CAS-01 cells showed that virus production increased from 12 hpi (log_10_ 3.2504 TCID_50_/ml), reaching a maximum titer of log_10_ 7.0000 TCID_50_/ml at 144 hpi. Taken together, these results suggest that cell-adapted ASFV can efficiently replicate in CAS-01 cells.

### Cytopathic and Hemadsorption (HAD) Properties of ASFV-Infected CAS-01 Cells

**Fig. 4:**
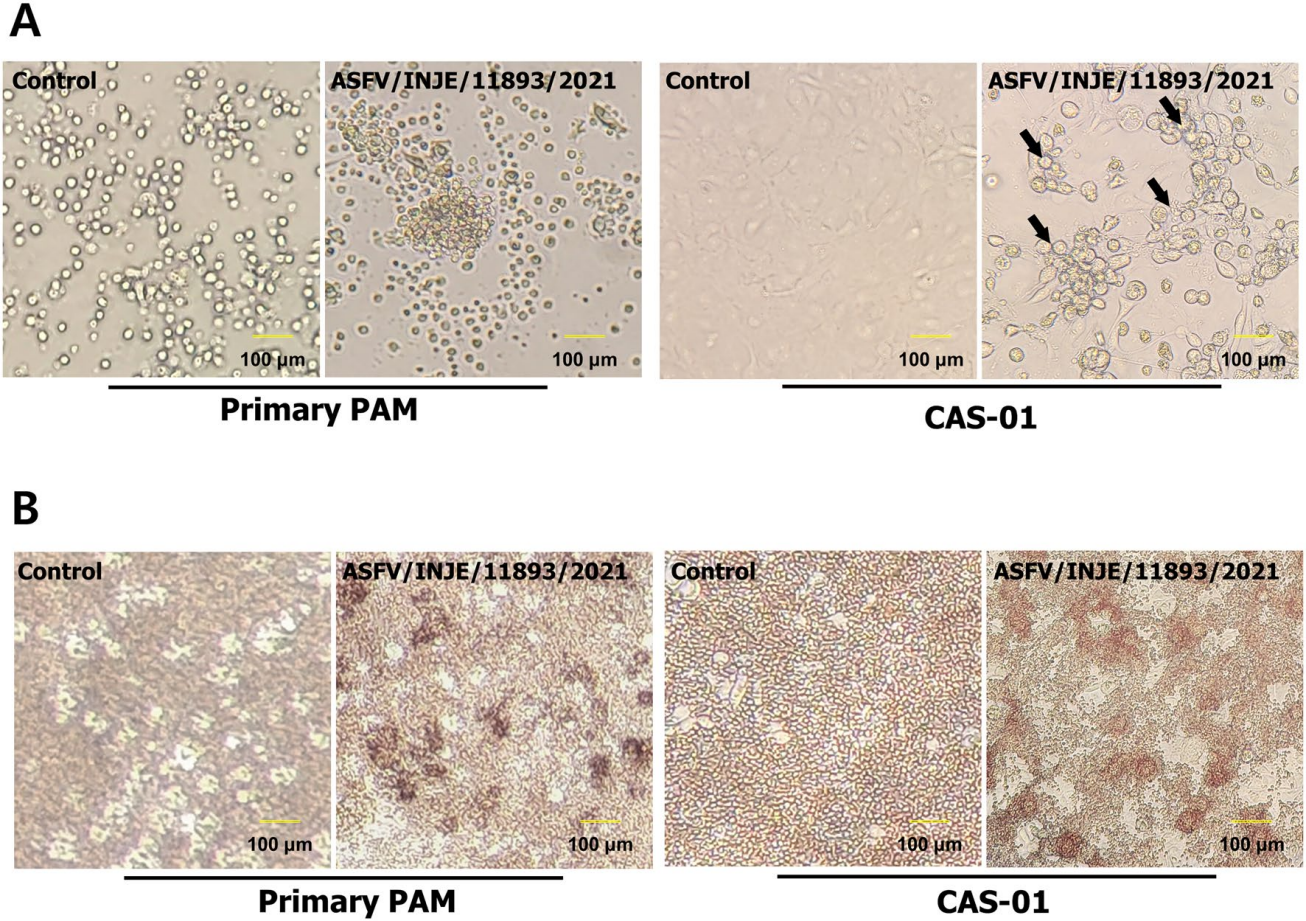
Cytopathic and hemadsorption (HAD) properties of ASFV/INJE/11893/2021-infected CAS-01 cells. **A** After 7 day post-infection (dpi), virus-induced cytopathic effect (CPE) was observed in Primary PAM and CAS-01. **B** After 2 h post-infection (hpi), 0.4% porcine red blood cell (RBC) was added and incubated for 7 days. Hemaadsorption (HAD) was checked every day for 7 days, and images were taken at 7 dpi. (Arrow in **A**, the right panel indicates the cytopathic effect upon ASFV infection). Data in **A** and **B** are representative of at least three independent experiments with two replicates, each with similar results

Further, to determine the infectivity of cell-passaged ASFV in CAS-01 cells, we observed its cytopathic effect and hemadsorption properties. As shown in Fig. 4A, ASFV-infected cells exhibited a prominent cytopathic effect (CPE) that was detectable at 6 days post-infection (dpi) in CAS-01 cells. However, ASFV-infected primary PAM cells have a round morphology, with massive vacuolization of the cytoplasm of cells that were detached from the culture plate. Therefore, proper CPEs could not be observed. In contrast, a significant CPE was observed in CAS-01 cells upon ASFV infection at the same multiplicity of infection (MOI). As indicated by the arrows, CAS-01 cells had a round morphology and formed clusters of cells. It is more relevant to evaluate the produced ASFV infective virus by the exploitation of a characteristic feature of the swine monocytes infection, which developed a rosette of erythrocytes around the infected cell. It is the basis of a conventional assay by “hemadsorption,” widely used both for diagnostic purposes and virus titration. As shown in Fig. 4B, hemadsorption was observed at 6 dpi in CAS-01 cells, similar to that in primary PAM cells. Our results demonstrate that cell-passaged ASFV-infected CAS-01 cells form rosettes with a phenotype very similar to the rosettes typically observed in ASFV-infected primary PAM cells.

## Discussion

Suitable in vitro systems for the detection, isolation, and manipulation of field isolates of pathogenic ASFV are only available for porcine primary macrophages and monocytes derived from peripheral blood or other tissues (Meloni et al., 2022). The quality of primary cell preparations may vary from batch to batch due to differences in the health of donor animals and preparation techniques, and the generation of primary macrophages is time-consuming and expensive and can result in contamination leading to cell waste (Gao et al., 2022). Moreover, using primary cells to produce large-scale vaccines is ethically challenging and, therefore, not feasible. Consequently, the development of safe and effective ASFV vaccines and diagnosis has been hampered by the lack of continuous cell lines suitable for ASFV isolation and propagation (Gao et al., 2022; Masujin et al., 2021). In light of these obstacles, the development of sustainable cell lines susceptible to ASFV infection is urgently needed.

In this study, we derived and validated highly ASFV-permissive CAS-01 cells capable of ASFV replication and passage. First, we isolated highly ASFV-permissive CAS-01 cell subpopulations from the MA-104 parental cell line by cell cloning. Second, we demonstrated stable replication and adaptation of isolated ASFV/INJE/11893/2021 and ASFV/INJE/13167/2021 over the serial passage, and we observed higher infectivity of the passaged virus than un-passaged ASFV in CAS-01 cells. Third, replication of passaged-ASFV in CAS-01 cells was confirmed by Ct value for ASFV DNA, HAD_50_/ml assay, TCID_50_/ml assay, and cytopathic effects and hemadsoption were observed similar to those in primary porcine cells. Taken together, our results strongly indicate that ASFV can be efficiently isolated and propagated using CAS-01 cells, which may be useful for the development of cell-adapted vaccines against ASFV.

ASFV isolation rates may vary with sample conditions, and the amount of virus at the beginning of passage is important for viral adaptation. Recently, the cell line MA-104 was used to identify infectious ASFV strains in clinical samples (Kwon et al., 2022; Rai et al., 2020). However, the parental MA-104 cell line has been shown to exhibit heterogeneous permissiveness to PRRS infection (Kim et al., 1993). Since we also observed similar results in a preliminary study of ASFV infection in MA-104 cells, the current study was designed to clone a highly ASFV-permissive homogenous cell population from the heterogeneous parental MA-104 cell line. As a result, we selected CAS-01 cells that exhibit significantly higher ASFV replication during cell passage than other cell clones.

Recent research efforts have focused on identifying and developing cell lines that support ASFV propagation and titration. WSL cells that express a high level of SLA 11 and SW3 exhibits susceptibility to either field isolates or laboratory-generated ASFV strains (Carrascosa et al., 2011; de León et al., 2013; Hernaez & Alonso, 2010). Attenuated ASFV NH/P68 has shown higher production in WSL cells compared to PAM cells (Sánchez et al., 2017a), but neither ASFV E70 nor Armenia/07 has shown efficient replication in the same cell line (Sánchez et al., 2017a, 2017b). Attenuated ASFV isolates Hinde and Uganda could infect to Immortalized Porcine Alveolar Macrophages (IPAM) (de León et al., 2013). However, a later Spanish research group reported that IPAM could not be infected with either the virulent ASFV Armenia/07 or the attenuated NH/P68 (Sánchez et al., 2017a, 2017b). In addition, the immortalized kidney macro-phage (IPKM) cell line has been demonstrated to be highly susceptible to ASFV infection, supporting the propagation of both virulent and cell-adapted ASFVs (Masujin et al., 2021) and porcine macrophage cell line Zuckerman macrophage-4 (ZMAC-4) has been reported to be susceptible to infection by eight different ASFV field isolates (Portugal et al., 2020). Especially, Plum Island porcine epithelial cells (PIPEC) cell line was engineered as a putative cell line for large-scale production of attenuated ASFV-G-ΔI177L (Borca et al., 2021). Additionally, monkey-derived cell lines such as Vero (Enjuanes et al., 1976), COS-1 (Hurtado et al., 2010), MS (Santurde et al., 1988; Tabares et al., 1987), CV1 (Sereda et al., 2022), MA104 (Oh et al., 2021; Rai et al., 2020), or MARC-145 (Kwon et al., 2022) and human-derived HEK293T (Keßler et al., 2018; Wang et al., 2021) cells have been studied for the adaptation and culture of virulent or cell-adapted ASFV strains. However, the practical application of these cells and specific characteristics, including genomic stability, of ASFV isolated and propagated from these cell lines require further study.

Serial passage of ASFV may lead to better adaptation and viral replication in ASFV field isolates. Preliminary studies have demonstrated that ASFV can be attenuated by serial passage in cell culture, suggesting the practical application of this technique for virology research, including vaccine development (Zhang et al., 2023). Due to repeated passages and cell adaptation in vitro, the viral genome can undergo genetic and phenotypic changes (Borca et al., 2021; Krug et al., 2015). ASFV that has adapted to being passaged in cells typically has prominent deletions in the genome, particularly in variable regions at both ends (Pires et al., 1997; Tabares et al., 1987). Generally, cell-adapted ASFV strains show decreased virulence and immunogenicity in swine (Sanford et al., 2016). However, some cases showed the same level of attenuation and better protective efficacy (Borca et al., 2021). The exact mechanism for the relationship between genomic alterations in cell-adapted ASFV and immunogenic characters remains unclear and requires further studies. However, this strategy is an essential methodology for the development of live attenuated ASFV vaccines for commercial production. In this study, ASFV/INJE/11893/2021 and ASFV/INJE/13167/2021 was continuously passaged in CAS-01 cells for 12 passages, and stable virus propagation and maintenance were confirmed. As reported in the previous literature (Pires et al., 1997; Tabares et al., 1987), prominent genome alterations are possible in ASFV viruses passaged in CAS-01 cells. To validate this critical hypothesis, our future investigations will focus on analyzing the entire genome sequence of passaged ASFV with the aim of verifying any genomic alterations that may occur during passage in CAS-01 cells and also performing vaccine studies including cellular, humoral immune response and protective efficacy following cell-passaged ASFV challenge in vivo conditions. Interestingly, upon cell passaged-ASFV infection, CAS-01 cells showed clear cytopathic effects, including rounded and formed clusters with a vacuolated cytoplasm compared with primary PAM cell infection. In addition, clear rosette formation was confirmed in CAS-01 cells through hemadsorption assay. These results suggest that CAS-01 cells are useful for ASFV isolation and virus passage. Moreover, growth characteristics of cell passaged-ASFV in CAS-01 cells showed high viral replication efficacy, similar to ASFV replication in the primary PAM cells.

In conclusion, we demonstrated that the CA-CAS-01-A (CAS-01) cell line is highly susceptible to ASFV infection and useful for the propagation of virulent ASFV strains. This cell line can be maintained in research laboratories or vaccine laboratories and exhibits numerous valuable properties for the isolation, replication, and adaptation of ASFV. Therefore, CAS-01 cells will be invaluable for advancing our understanding of ASFV and developing technologies to combat it, such as live-attenuated vaccines.

## Data Availability

The data supporting the findings in this study are available from the corresponding author upon reasonable request.
